# Detection Performance Improvement of Distributed Vibration Sensor Based on Curvelet Denoising Method

**DOI:** 10.3390/s17061380

**Published:** 2017-06-14

**Authors:** Zengguang Qin, Hui Chen, Jun Chang

**Affiliations:** School of Information Science and Engineering and Shandong Provincial Key Laboratory of Laser Technology and Application, Shandong University, Jinan 250100, China; hui931001@163.com (H.C.); changjun@sdu.edu.cn (J.C.)

**Keywords:** vibration sensing, phase-sensitive optical time domain reflectometry, curvelet denoising, signal-to-noise ratio

## Abstract

A curvelet denoising method has been proposed to reduce the time domain noise to improve the detection performance in the distributed fiber vibration sensing system based on phase-sensitive optical time domain reflectometry. The raw Rayleigh backscattering traces are regarded as a gray image and the random noise can be eliminated by the curvelet transform; hence, the amplitude difference induced by the external vibration can be extracted. The detection of a vibration event with 10 m spatial resolution along a 4 km single mode fiber is demonstrated. The signal-to-noise ratio (SNR) of location information for 50 Hz and 1 kHz vibration based on this new method increases to as high as 7.8 dB and 8.0 dB, respectively, compared to the conventional method, showing the remarkable denoising capability of this new approach.

## 1. Introduction

Fiber-optical vibration sensors have attracted significant interest in recent years owing to their immunity to electromagnetic interference, high detection sensitivity, fast response, and low cost. In general, fiber-optical vibration sensors can be categorized into two main types: point and distributed sensors. Point sensors such as fiber Bragg gratings (FBG) [1,2] and Fabry–Perot interferometers [3] can realize vibration measurement with high resolution and wide bandwidth. However, the point sensors require a prior knowledge of potential fault location in the practical applications. Distributed sensors provide spatially-resolved measurement capability. A large variety of interferometric architectures for distributed vibration sensing, including the Sagnac interferometer, dual Mach–Zehnder interferometer, and Sagnac interferometer merged with Mach–Zehnder interferometer [4,5,6], have been proposed and demonstrated. Although this kind of sensor has high sensitivity and wide frequency response range, the spatial resolution is usually poor. Moreover, distributed vibration sensors based on the scattering effects inside the optical fiber have been demonstrated, which includes polarization optical time domain reflectometry (P-OTDR) [7], phase-sensitive OTDR (φ-OTDR) [8,9,10,11,12,13], and Brillouin scattering-based sensors [14,15]. As one of the most promising technologies for distributed vibration sensing, φ-OTDR has attracted considerable attention in recent years due to their high spatial resolution, broad frequency response range, and multiple events detection capability.

In φ-OTDR system, optical pulses from a highly coherent light source are launched into a sensing fiber and the Rayleigh backscattering trace is modulated in the form of speckle-like profile because of the coherent interaction of multiple scattering centers within the pulse duration [16]. Truly distributed vibration sensing can be realized by analyzing the variation of the traces induced by the refractive index change around the vibration location. Nevertheless, the Rayleigh backscattering signal is very weak and easily influenced by the environment. Moreover, position-dependent signal fading caused by modulation instability will also affect the performance of the φ-OTDR system [17] if the pulse peak power is too high. Meanwhile, the background noise varies due to many uncertain sources (e.g., laser phase noise and environmental temperature fluctuation), which causes the signal-to-noise ratio (SNR) to be quite low and makes the vibration hardly distinguishable. Many studies have been performed seeking to suppress the random noise and enhance the SNR. A distributed fiber intrusion sensor using a narrow linewidth and low frequency shift laser based on φ-OTDR has been demonstrated [18]. Location information of intrusion is retrieved with a relatively low SNR by subtracting the Rayleigh backscattering traces from the earlier stored traces with 10 averaging time. Moving average and moving differential method is proposed in another φ-OTDR system using coherent detection in order to moderate the random noise and highlight the changes among traces [19]. The SNR of position information is increased, but the frequency response of the system decreases appreciably. A wavelet denoising method which provides a powerful capability to eliminate the random noise in the φ-OTDR system has been adopted [20]. A system with frequency response from 20 Hz to 8 kHz and 0.5 m spatial resolution over 1 km sensing fiber has been realized. However, the results of the wavelet denoising method are related to the basic function of wavelet transform, which needs to be optimized.

All of the aforementioned methods are mainly focused on improving the system performance by reducing the random noises restricted to the one-dimensional (1D) raw traces. Performance enhancement of the φ-OTDR system is obtained without exploiting the redundancies and correlations included in the two-dimensional domain of the raw data. Considering the feature of distributed fiber vibration sensor based on the φ-OTDR system, the acquired data contains repeated structures of information in a two-dimensional (2D) domain (time and position), which means that the SNR of the location information can be improved by processing the raw data in a two-dimensional way [21].

In this paper, a novel efficient denoising method based on curvelet transform is demonstrated to improve the performance of the φ-OTDR system for the first time. A two-dimensional image consisting of Rayleigh traces is regarded as the processing object instead of the usual 1D array of the data. The random noises can be suppressed efficiently by the curvelet denoising method with an optimized Monte Carlo thresholding rule. Experimental results show that an SNR of location information as high as 7.8 dB and 8.0 dB compared to conventional method for 50 Hz and 1 kHz vibration events can be obtained in a single mode fiber with 4 km length when pulse width is 100 ns. This new curvelet denoising method shows great potential in retrieving the vibration position precisely under the condition of strong background noise in a distributed vibration sensor with a φ-OTDR system.

## 2. Curvelet Denoising Method

Curvelet transform is a multiscale method which was developed in the last few years and has been used for image denoising with a good performance [20]. The form of the continuous-time curvelet transform is shown as below:

$$c\left(j,l,k\right)=\int_{R^{2}}f\left(x\right)\bar{\varphi_{j,l,k}\left(x\right)}dx$$

where *j*,*l*,*k* are the scale parameter, orientation parameter, and spatial location parameter, respectively. $\bar{\varphi_{j,l,k}\left(x\right)}$ is the conjugate function of the curvelet function. *R*^2^ represents the two-dimensional space. *c*(*j*,*l*,*k*) is the curvelet coefficient.

In order to make the curvelet transform easier to use and understand, the fast discrete curvelet transform (FDCT) is proposed and is considerably simpler. The expression is given as:

$$c^{D}\left(j,l,k\right)=\underset{t_{1},t_{2}}{\sum }f\left[t_{1},t_{2}\right]\bar{\varphi_{j,l,k}^{D}\left[t_{1},t_{2}\right]}$$

where each $\varphi_{j,l,k}^{D}$ is a digital curvelet waveform.

There are two means by which to implement FDCT; namely, via wrapping-based transform and via unequally-spaced fast Fourier transform (USFFT). The difference between the two manipulations is that curvelet is translated in different ways at a given scale and angle. Concretely, in all quadrants, the translation grid for each angle is the same in the wrapping-based transform, while it is in line with the direction of the curvelet in USFFT. Therefore, the wrapping-based transform is easier to be comprehended and applied. A table of digital curvelet coefficients indexed by the *j*,*l*,*k* can be obtained by FDCT. Curvelet coefficients are divided into multiple scale layers, and the first layer is named the “coarse layer”, which is made up of low-frequency coefficients. The outermost layer is called the “fine layer”, and consists of high-frequency coefficients.

In a φ-OTDR system, the data can be processed as a grey image which is formed by Rayleigh backscattering traces. Actually, the image is a two-dimensional matrix, and the matrix row represents the location information while the column represents the change of light intensity. The curvelet denoising method applied in the φ-OTDR system includes the following steps: (1) collect Rayleigh backscattering traces and constitute a two-dimensional matrix; (2) apply FDCT to the matrix and obtain curvelet coefficients; (3) choose an optimized thresholding to remove noise components form the curvelet coefficients; (4) restore the image with the inverse FDCT.

Generally, the Monte Carlo method [22] is used for thresholding in curvelet denoising, and the hard-thresholding rule for curvelet denoising can be defined as:

$$T=\{\begin{array}{c} kee_{j}\;\mathrm{if}\;\left|T\right|\geq kee_{j}\;\\ 0\;\mathrm{if}\;\left|T\right|\leq kee_{j}\; \end{array},$$

In the equation, *T* represents the noisy curvelet coefficients, *k* is the scale value which is a constant, *e* is the standard deviation of noise, and *e_j_* is the standard deviation of coefficients for every scale generated by white Gaussian noise with a mean value of zero and a variance value of one via curvelet transform under Monte Carlo simulation. The curvelet coefficients of the noise can be eliminated by the above threshold step, since the noises embedded in the raw data usually correspond to the curvelet coefficients with small absolute value. However, there are still some redundant noises contained in the denoised curvelet coefficients according to the energy distribution property of curvelet coefficients. Therefore, the performance of denoising needs to be further optimized by varying the scale value *k*. An optimized scale value is proposed, and the formula is as follows:

$$k=2^{\frac{1-j}{2}}\sqrt{2\log_{2}\left(\frac{L_{j}}{J}\right)}$$

where *J* is the total number of scale layers. *j* = 1,…, *J*. *L_j_* is the number of angles for every scale layer. The value of *k* will be various with respect to different scale layers.

## 3. Experimental Setup and Results

**Figure 1:**
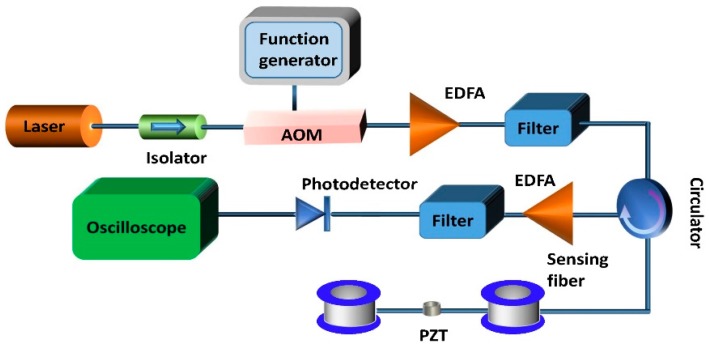
Experimental setup of a phase-sensitive optical time domain reflectometry (φ-OTDR) system using direct detection. AOM: acoustic optical modulator; EDFA: erbium-doped fiber amplifier.

The experimental setup of the φ-OTDR system is shown in Figure 1. An external cavity laser (ECL) with narrow linewidth of less than 10 kHz and maximum output power of 10 mW was used as the light source. Optical pulses were generated by an acoustic optical modulator (AOM) driven by an arbitrary function generator. An erbium-doped fiber amplifier (EDFA) was utilized to amplify the pulses, and the spontaneous emission noise was removed by a tunable fiber Bragg grating filter. Then, the pulses were launched into the fiber under test via an optical circulator and the Rayleigh backscattering light was amplified by using another EDFA. The output of the EDFA was filtered by another FBG to eliminate the amplified spontaneous emission (ASE) noise. Then, the Rayleigh backscattering light was detected by the photo detector (PD) and the electric signals were collected by a high-speed oscilloscope.

A PZT cylinder with 1 m single mode fiber wound was put at the end of the 4 km sensing fiber in our system as a vibration source. The PZT cylinder was driven by a function generator, and its vibration frequency could be adjusted from several Hz to kHz. The fiber was fixed on the surface of the PZT cylinder by instant glue, and the vibration of the PZT could be transmitted to the fiber. In our experiment, the pulse width was set to be 100 ns and the repletion rate was 10 kHz. One thousand traces were collected by a high-speed oscilloscope with 100 MHz sampling rate, and the total data acquisition time was 0.1 s.

**Figure 2:**
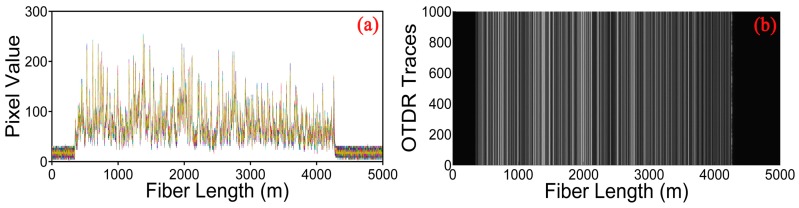
(**a**) Location information constituted by consecutive raw Rayleigh backscattering curves for 50 Hz vibration event; (**b**) A gray image constituted by consecutive Rayleigh curves.

Figure 2a shows 1000 consecutive raw Rayleigh backscattering traces for a 50 Hz vibration event. We can find that the Rayleigh backscattering curves show a jagged appearance because of the coherent superposition of the light from multiple scattering centers along the fiber within the optical pulse duration. However, the position of the vibration can hardly be determined from these raw traces, since the data is deteriorated by the random noise. Figure 2b shows a gray image constituted by the above 1000 Rayleigh backscattering curves. The gray value is proportional to the amplitude of the Rayleigh traces. The brightness of the image varies along the fiber with the magnitude of the Rayleigh signals alterations. A weak periodic change of pixel value can be observed at the end of the fiber under test, representing the position of vibration. However, the extraction of vibration information is not good enough and may lead to false alarms in the real applications. Hence, curvelet denoising method is adopted here to suppress the random noise.

**Figure 3:**
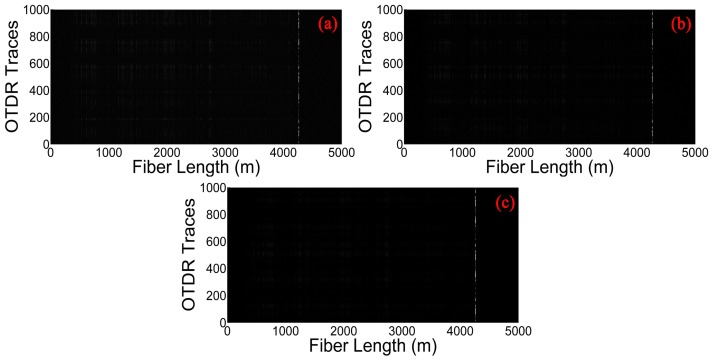
Gray image obtained by different methods. (**a**) Moving differential method; (**b**) Curvelet denoising method based on the traditional thresholding; (**c**) Curvelet denoising method based on the optimized thresholding.

**Figure 4:**
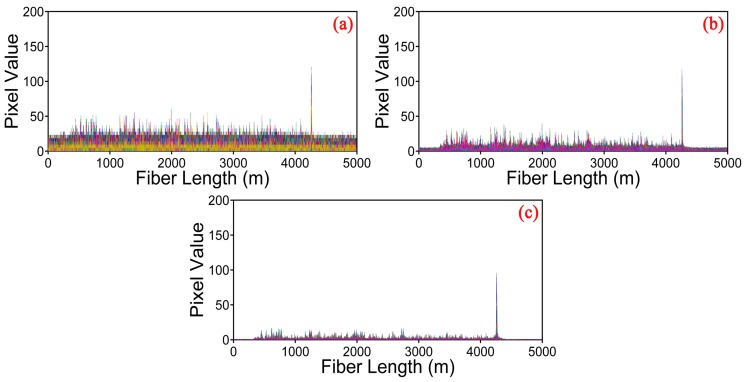
Location information under different methods. (**a**) Moving differential method; (**b**) Curvelet denoising method based on the traditional thresholding; (**c**) Curvelet denoising method based on the optimized thresholding.

We employ the moving differential method [19], which subtracts the rows with its moving reference to determine the position of vibration, shown in Figure 3. The position information of Figure 3a is directly obtained by moving differential method. Figure 3b,c gives the results of the raw data after processing by the curvelet denoising method based on the traditional thresholding and the optimized thresholding, respectively. The vibration location is represented by the bright line at the end of the sensing fiber. According to these three figures, we can find that the grey image obtained by the optimized thresholding method has the lowest noise background. In order to observe the improvement of the proposed curvelet denoising method clearly and visually, the location information of the vibration obtained by different methods are shown in Figure 4a–c. The SNR of the location information is defined as SNR = 10 × log10(V_signal_/V_noise_), interpreted as a ratio of the signal peak voltage to the background noise [19]. Here, the voltage is converted to a gray value within the range of 0–255. In Figure 4a, the pixel value of signal and noise is equal to 120.54 and 60.25 for moving differential method respectively, so the SNR is equal to 3.0 dB. In Figure 4b, when applying the traditional thresholding, the SNR is 4.7 dB. In Figure 4c, the SNR of the location improves to 7.8 dB under the optimized thresholding. It is obvious that the SNR of location information is significantly increased by the curvelet transform with optimized thresholding.

**Figure 5:**
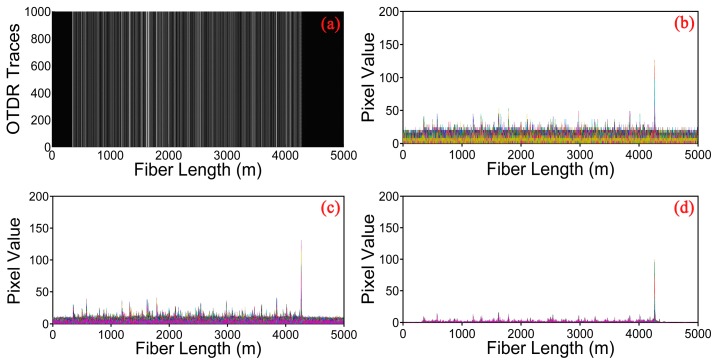
Experiment results of 1 kHz vibration event. (**a**) A gray image constituted by consecutive Rayleigh curves; (**b**) Moving differential method; (**c**) Curvelet denoising method based on the traditional thresholding; (**d**) Curvelet denoising method based on the optimized thresholding.

Figure 5a shows a gray image constituted by the 1000 Rayleigh backscattering curves for 1 kHz vibration event. When compared with the gray image of 50 Hz, we can see that a distinct periodic change of pixel value appears at the end of the fiber. The location information processed by different methods is shown in Figure 5b–d. Similarly, the noise is removed by employing the curvelet denoising method and the image obtained by the optimized thresholding displays a remarkable effect in signal denoising.

**Figure 6:**
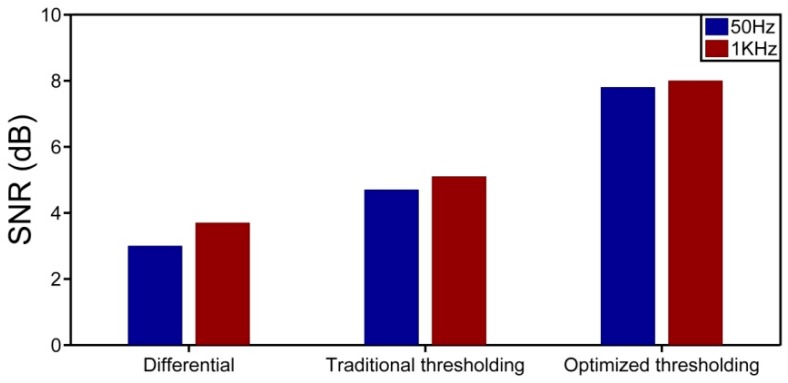
SNR of location information for the vibration events with frequency 50 Hz and 1 kHz based on different methods.

In order to observe the denoising effect intuitively, the SNR for 50 Hz and 1 kHz using different methods is shown in Figure 6. In both cases, compared with the moving differential method, it is evident that the SNR of location information is enhanced by the curvelet denoising method. Meanwhile, with the comparison of two types of thresholding, the optimized thresholding is better in terms of noise reduction.

**Figure 7:**
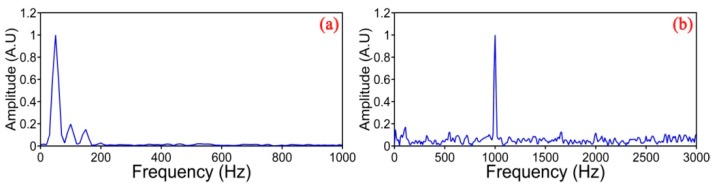
Frequency information for the vibration events. (**a**) For 50 Hz; (**b**) For 1 KHz.

Figure 7 shows the normalized power spectra for the vibration events of 50 Hz and 1 KHz, respectively, by the Fourier transform at the vibration location.

**Figure 8:**
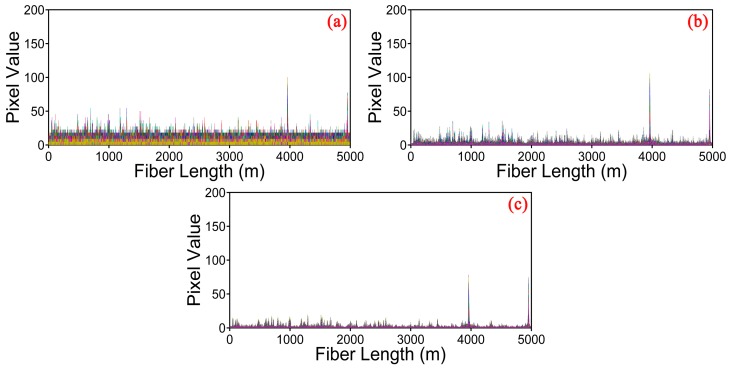
Location information for two PZT vibrations detection under different methods. (**a**) Moving differential method; (**b**) Curvelet denoising method based on the traditional thresholding; (**c**) Curvelet denoising method based on the optimized thresholding.

We also investigate the detection capability of the φ-OTDR system for multiple vibration events by using curvelet denoising method. Two PZT cylinders were used as the vibration sources along 5 km sensing fiber, and both were driven by a 200 Hz sinusoid wave. The results of simultaneous measurement under moving differential method, traditional thresholding, and optimized thresholding are shown in Figure 8a–c, respectively. Two clear peaks at 3960 m and 4950 m corresponding to the positions of vibration events can be observed for the three methods. It is obvious that the SNR of the location is significantly improved by curvelet denoising method with optimized thresholding compared to the other two methods.

## 4. Conclusions

In this paper, we introduced a novel curvelet denoising method to improve the detection performance of φ-OTDR system; FDCT is applied on the image composed of Rayleigh traces, and a series of curvelet coefficients is obtained. An optimized thresholding based on Monte Carlo threshold rule is proposed to optimize the denoising results. The SNR of the location information of vibration event is discussed by different signal processing methods and thresholding rules. An SNR of location for 50 Hz and 1 kHz vibration events as high as 7.8 dB and 8.0 dB can be achieved, respectively, which proves that the curvelet denoising method is an effective tool to improve the performance of φ-OTDR systems.
